# Association Between the Combined Effects and Joint Trajectories of Depression and Frailty and the Risk of Digestive Diseases: A Longitudinal Study

**DOI:** 10.1002/brb3.70877

**Published:** 2025-09-21

**Authors:** Xiaofei Fan, Xiaoming Qiao, Qianqian Wu, Tao Han

**Affiliations:** ^1^ Shandong Medical College Jinan Shandong China; ^2^ Community Management Department the Fourth People's Hospital of Jinan Jinan Shandong China

**Keywords:** depression | digestive diseases | frailty | gastrointestinal diseases | liver diseases

## Abstract

**Background and Aims:**

Digestive diseases, including gastrointestinal and liver disorders, are prevalent and contribute significantly to morbidity, healthcare costs, and reduced quality of life, particularly among middle‐aged adults. Frailty, characterized by reduced physiological reserve, and depression, a common psychological disorder, have both been linked to an increased risk of these conditions. However, the combined impact of frailty and depression on digestive diseases remains poorly understood. This study aimed to examine the combined effects and joint trajectories of frailty and depression on the risk of digestive diseases in middle‐aged adults and explore potential mechanisms of their interaction.

**Methods:**

We conducted a prospective cohort study using data from the China Health and Retirement Longitudinal Study, involving 7876 participants aged 45 and above. Participants were classified into three frailty categories (robust, pre‐frail, frail) and assessed for depression. The incidence of digestive, gastrointestinal, and liver diseases was monitored over a median follow‐up of 9 years. We utilized Cox regression models to evaluate the individual and joint effects of frailty and depression on disease risk.

**Results:**

During follow‐up, 2076 participants developed new digestive system diseases (26.36% incidence). Frailty and depression were independently associated with increased risks of digestive (hazard ratio [HR] for frail = 2.00, HR for depression = 1.61), gastrointestinal (HR for frail = 2.08, HR for depression = 1.67), and liver diseases (HR for frail = 2.14, HR for depression = 1.31). No significant multiplicative or additive interactions between the two were observed. Mediation analysis indicated that depression mediated 17.4% of the relationship between frailty and digestive diseases, whereas frailty mediated 18.8% of the relationship between depression and digestive diseases. The combined frailty and depression index improved predictive accuracy for all disease outcomes. In the joint trajectory analysis, the group with consistently higher depression scores and faster increases in the frailty index had the highest risk for all diseases.

**Conclusions:**

Integrated assessment of frailty and depression should be prioritized in residual risk stratification and primary prevention strategies for digestive diseases.

## Graphical abstract

This study identifies the compounded impact of frailty and depression on the risk of digestive diseases, revealing that worsening frailty and depression significantly elevate the risk for gastrointestinal and liver disorders. Integrated assessments of frailty and depression may enhance early identification and intervention strategies for digestive health.

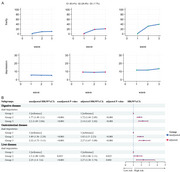

**Abbreviations**
AUC: area under the curve
AP: attributable proportion
BMI: body mass index
CESD: Center for Epidemiologic Studies Depression Scale
CHARLS: China Health and Retirement Longitudinal Study
CIs: confidence intervals
DCA: decision curve analysis
FI: frailty index
HbA1c: glycated hemoglobin
HR: hazard ratio
IDI: integrated discriminant improvement index
ICR: interaction contrast ratio
LDL‐C: low‐density lipoprotein cholesterol
NDE: natural direct effect
NIE: natural indirect effect
NRI: net reclassification index
ROC: receiver operating characteristic
RERI: relative excess risk due to interaction
RCS: restricted cubic spline
SI: synergy index
TyG: triglyceride‐glucose

## 1 Introduction

Digestive diseases are a significant public health concern worldwide, contributing to substantial morbidity, healthcare costs, and reduced quality of life (Diseases and Injuries 2020; Peery et al. 2012; Quigley et al. 2013). These conditions, encompassing gastrointestinal and liver diseases, often result from complex interactions between physiological, psychological, and lifestyle factors (Wu et al. 2024; Njei et al. 2024). Among the middle‐aged population, these diseases are particularly burdensome. Identifying modifiable risk factors and understanding their interplay is therefore essential for devising effective prevention and management strategies.

Frailty, a clinical syndrome characterized by diminished physiological reserve and increased vulnerability to adverse health outcomes, has emerged as a key determinant of various chronic conditions, including digestive diseases (Bellelli et al. 2024; He et al. 2024). Frailty reflects a cumulative decline in multiple bodily systems and has been associated with inflammation, impaired gut motility, and dysbiosis, which may predispose individuals to gastrointestinal and liver diseases (Sealy et al. 2024; Naito et al. 2024).

Depression, another prevalent condition among middle‐aged adults, has similarly been linked to digestive diseases (Santomauro et al. 2024; Zhou et al. 2024). As a multifaceted psychological disorder, depression can influence digestive health through neuroendocrine dysregulation, inflammation, and lifestyle factors such as poor diet and physical inactivity (Miller and Raison 2016; Tobaruela‐Resola et al. 2024). Notably, depression and frailty frequently coexist. Despite their individual associations with digestive diseases, the combined impact of frailty and depression remains poorly understood.

Furthermore, although prior studies have explored the independent effects of frailty and depression on chronic diseases, few have examined their joint effects or potential mediating mechanisms in the context of digestive diseases. Investigating whether frailty mediates the impact of depression—or vice versa—on digestive, gastrointestinal, and liver diseases could provide valuable insights into shared biological and psychosocial pathways.

This research seeks to address critical knowledge gaps by utilizing data from the nationally representative China Health and Retirement Longitudinal Study (CHARLS) cohort. The specific objectives are to: (1) examine the independent and joint effects of frailty and depression on the incidence of digestive diseases; (2) investigate interaction and mediation between frailty and depression; (3) assess the predictive utility of frailty and depression for identifying individuals at elevated risk; and (4) examine the impact of joint trajectories of frailty and depression on disease. By elucidating the complex relationships between frailty, depression, and digestive health, this study aims to inform the development of preventions for digestive diseases.

## 2 Methods

### 2.1 Study Participants

This study draws upon data from the CHARLS. Conducted by the National Development Research Institute at Peking University, CHARLS spans five waves from 2011 to 2020. Participants from the inaugural wave in 2011 (*n* = 17,708) were designated as the baseline cohort, with subsequent follow‐ups in 2013, 2015, 2018, and 2020. Following exclusions for incomplete baseline exposure or digestive disease data, age under 45, lack of follow‐up responses, or a history of digestive diseases at baseline, the final analytical sample included 7876 individuals (Figure S1). The CHARLS protocol received approval from the Biomedical Ethics Review Board at Peking University (IRB00001052‐11015), with all participants providing informed consent. All study procedures adhered to the ethical principles of the Declaration of Helsinki.

### 2.2 Assessment of Frailty Index (FI) and Depression

Frailty is characterized by the progressive accumulation of age‐associated health deficits. Utilizing the CHARLS dataset, we identified 31 variables to develop the FI. These variables encompassed chronic illnesses, physical functioning, disabilities, and cognitive performance (Table S1) (He et al. 2023). Each variable was dichotomized as 0 or 1, except for item 31, which assessed cognitive functioning. For this item, 0 represented no impairment, whereas 1 denoted impairment. As a continuous variable, item 31 ranged from 0 to 1, with higher scores indicating greater cognitive dysfunction. The 31‐item FI (31‐FI) was computed for each participant by dividing the total number of health deficits by 31. Consequently, the 31‐FI is a continuous metric ranging from 0 to 1, with higher values signifying greater frailty. Frailty status was categorized into three groups: robust (FI ≤ 0.10), pre‐frail (0.10 < FI < 0.25), and frail (FI ≥ 0.25) (He et al. 2024).

Depression score was assessed using the Center for Epidemiologic Studies Depression Scale (CESD), a 10‐item instrument with each item scored on a 3‐point scale, yielding a total score range of 0–30. A CESD score exceeding 10 was classified as indicative of depressive symptoms (Wang et al. 2024).

### 2.3 Outcome Ascertainment

The primary outcome of the study was the incidence of stroke. Following the approach of prior research, gastrointestinal and liver diseases were identified through standardized questions: “Have you been diagnosed with a gastrointestinal disease (excluding tumors or cancer) by a doctor?” and “Have you been diagnosed with liver disease (excluding fatty liver, cancer, or tumors) by a doctor?” Participants who responded “Yes” to either question were classified as having gastrointestinal or liver diseases (Chen et al. 2024).

### 2.4 Covariates

Covariates included gender, baseline age, marital status (categorized as “married” and “other”), and educational attainment, which was divided into three categories: “primary and below,” “secondary,” and “university” based on years of education. Place of residence was classified as either “urban” or “rural.” Current smoking and drinking statuses, physical activity, kidney disease, dyslipidemia, participation in social activities, and living alone were all binary variables, categorized as either “yes” or “no.” Additionally, covariates included the triglyceride‐glucose (TyG) index, calculated using the following formula: ln(TG [mg/dL] × FBG [mg/dL]/2), as well as glycated hemoglobin (HbA1c) (%) and low‐density lipoprotein cholesterol (LDL‐C) (mg/dL) (5). Body mass index (BMI) categorization included four groups: underweight (<18.5), normal weight (18.5–23.9), overweight (24–24.9), and obesity (≥25).

### 2.5 Statistical Analysis

In this study's descriptive statistics, baseline characteristics across frailty and depression groups were compared using ANOVA, chi‐square tests, or Kruskal–Wallis rank‐sum tests, depending on data distribution. Means and standard deviations were reported for continuous variables, whereas percentages were calculated for categorical data. Multivariable‐adjusted Cox proportional hazards models were applied to estimate risk ratios and 95% confidence intervals (CIs) for digestive diseases associated with frailty and depression. Univariate analyses utilized unadjusted models, whereas adjusted models included all covariates. Missing covariate data were addressed using multiple imputations with chained equations.

To analyze the combined effect of depression and frailty on disease risk, participants were grouped into six categories based on the presence or absence of these factors. Using the reference group (first quartile without depression and frailty), hazard ratios (HRs) and 95% CIs for stroke incidence were calculated for the remaining groups. Dose–response and linear relationships across these groups were examined using 3‐node restricted cubic spline (RCS) curves.

The interaction between depression and frailty concerning digestive disease risk was explored through both additive and multiplicative interaction models. For multiplicative interactions, a product term for depression and frailty was included in the Cox model, with HRs and 95% CIs used to evaluate significance. Additive interactions were assessed using metrics derived via the delta method: interaction contrast ratio (ICR) or relative excess risk due to interaction (RERI), attributable proportion (AP), and synergy index (SI).

Mediating effects of depression and frailty were analyzed using the “mets” software package, focusing on dichotomous variables and including only robust and frail groups (include the frail group and pre‐frail group). Direct and indirect mediation effects were examined through regression models. The natural direct effect (NDE) represented the variable's direct impact on disease risk, whereas the natural indirect effect (NIE) quantified the mediated effect. The mediation ratio was computed as log(NIE)/(log(NIE) + log(NDE)).

Predictive performance of various metric combinations was evaluated with receiver operating characteristic (ROC) curves. Decision curve analysis (DCA) assessed the net clinical benefit, and incremental predictive value differences between groups were tested using the net reclassification index (NRI) and the integrated discriminant improvement index (IDI).

Sensitivity analyses were conducted to validate the findings and investigate potential variations:

1. Stratified analyses were performed on the basis of sex (male and female) and age (<60 and ≥60 years).
2. The analyses were repeated using the original, unmodified dataset.
3. Mediation analyses focused on the frailty and robust groups to evaluate intermediary effects between frailty levels and depression.
4. The added predictive value of incorporating the FI and depression levels into traditional risk factors was examined.
5. *E* values were calculated to assess the influence of potential unmeasured confounders on the study's outcomes.

All analyses were carried out using R (version 4.2.2) and Stata/MP 18.0, with data cleaning conducted in Stata and statistical modeling performed in R. Mediation analysis was handled using the “mets” package, whereas Cox regressions were run with the “survival” package. Interaction effects, both additive and multiplicative, were assessed using the “interactionR” package. The “rcssci” package was used for creating RCS curves, and ROC curves were generated with the “rms” package. Metrics such as NRI and IDI were calculated via the “survIDINRI” package.

## 3 Results

### 3.1 Population Characteristics

At baseline, a total of 7876 participants were included and categorized into three groups based on frailty levels: 3265 were classified as robust, 3966 as pre‐frail, and 645 as frail. The mean age of participants was 57 years, with 4031 (51.18%) being male. Compared to those with lower frailty levels, participants with higher frailty levels were more likely to be older, female, exhibit depressive characteristics, and have a higher prevalence of digestive system‐related diseases during follow‐up. Baseline characteristics stratified by frailty levels are presented in Table 1, whereas characteristics stratified by depression status are detailed in Table S2.

**TABLE 1:** Baseline characteristics of participants.

Groups	Robust	Pre‐frail	Frail	Overall	*p*
	(*N* = 3265)	(*N* = 3966)	(*N* = 645)	(*N* = 7876)	
**Gender, *n*(%)**					<0.001
Male	1856 (56.85%)	1908 (48.11%)	267 (41.40%)	4031 (51.18%)	
Female	1409 (43.15%)	2058 (51.89%)	378 (58.60%)	3845 (48.82%)	
**Age**	55.00 [49.00;61.00]	58.00 [52.00;65.00]	63.00 [57.00;70.00]	57.00 [51.00;64.00]	<0.001
**Depression scores**	4.00 [2.00;8.00]	7.00 [3.00;11.00]	12.00 [7.00;17.00]	6.00 [3.00;11.00]	<0.001
**Depression**					<0.001
No	2727 (83.52%)	2591 (65.33%)	240 (37.21%)	5558 (70.57%)	
Yes	538 (16.48%)	1375 (34.67%)	405 (62.79%)	2318 (29.43%)	
**HbA1c (%)**	5.10 [4.80;5.42]	5.20 [4.89;5.50]	5.20 [4.90;5.60]	5.10 [4.80;5.50]	<0.001
**LDL‐C (mg/dL)**	114.82 [93.18;135.97]	115.21 [93.56;138.70]	113.27 [93.94;136.08]	114.82 [93.56;137.24]	0.216
**TyG index**	8.60 [8.20;9.04]	8.68 [8.26;9.13]	8.75 [8.38;9.21]	8.65 [8.23;9.10]	<0.001
**Marital status**					<0.001
Other	262 (8.02%)	477 (12.03%)	111 (17.21%)	850 (10.79%)	
Married	3003 (91.98%)	3489 (87.97%)	534 (82.79%)	7026 (89.21%)	
**Educational level**					<0.001
Less than upper secondary education	2677 (81.99%)	3471 (87.52%)	607 (94.11%)	6755 (85.77%)	
Upper secondary	498 (15.25%)	422 (10.64%)	32 (4.96%)	952 (12.09%)	
Tertiary education	90 (2.76%)	73 (1.84%)	6 (0.93%)	169 (2.15%)	
**Residence**					<0.001
Urban	1397 (42.79%)	1611 (40.62%)	226 (35.04%)	3234 (41.06%)	
Rural	1868 (57.21%)	2355 (59.38%)	419 (64.96%)	4642 (58.94%)	
**Smoking status**					<0.001
No	2058 (63.03%)	2766 (69.74%)	500 (77.52%)	5324 (67.60%)	
Yes	1207 (36.97%)	1200 (30.26%)	145 (22.48%)	2552 (32.40%)	
**Drinking status**					<0.001
No	1911 (58.53%)	2627 (66.24%)	500 (77.52%)	5038 (63.97%)	
Yes	1354 (41.47%)	1339 (33.76%)	145 (22.48%)	2838 (36.03%)	
**Physical activity**					0.224
No	335 (10.26%)	422 (10.64%)	81 (12.56%)	838 (10.64%)	
Yes	2930 (89.74%)	3544 (89.36%)	564 (87.44%)	7038 (89.36%)	
**BMI categorization**					<0.001
Underweight less than 18.5	333 (10.20%)	415 (10.46%)	77 (11.94%)	825 (10.47%)	
Normal weight from 18.5 to 23.9	1290 (39.51%)	1344 (33.89%)	225 (34.88%)	2859 (36.30%)	
Overweight from 23 to 24.9	619 (18.96%)	747 (18.84%)	80 (12.40%)	1446 (18.36%)	
Obesity from 25 to 100	1023 (31.33%)	1460 (36.81%)	263 (40.78%)	2746 (34.87%)	
**Chronic kidney diseases**					<0.001
No	3182 (97.46%)	3795 (95.69%)	589 (91.32%)	7566 (96.06%)	
Yes	83 (2.54%)	171 (4.31%)	56 (8.68%)	310 (3.94%)	
**Dyslipidemia**					<0.001
No	3099 (94.92%)	3513 (88.58%)	530 (82.17%)	7142 (90.68%)	
Yes	166 (5.08%)	453 (11.42%)	115 (17.83%)	734 (9.32%)	
**Social activity**					<0.001
No	1553 (47.57%)	2016 (50.83%)	367 (56.90%)	3936 (49.97%)	
Yes	1712 (52.43%)	1950 (49.17%)	278 (43.10%)	3940 (50.03%)	
**Live alone**					0.004
No	3123 (95.65%)	3760 (94.81%)	597 (92.56%)	7480 (94.97%)	
Yes	142 (4.35%)	206 (5.19%)	48 (7.44%)	396 (5.03%)	
**Incident digestive diseases**					<0.001
No	2578 (78.96%)	2804 (70.70%)	418 (64.81%)	5800 (73.64%)	
Yes	687 (21.04%)	1162 (29.30%)	227 (35.19%)	2076 (26.36%)	
**Incident gastrointestinal diseases**					<0.001
No	2693 (82.48%)	2988 (75.34%)	444 (68.84%)	6125 (77.77%)	
Yes	572 (17.52%)	978 (24.66%)	201 (31.16%)	1751 (22.23%)	
**Incident liver diseases**					<0.001
No	3101 (94.98%)	3652 (92.08%)	581 (90.08%)	7334 (93.12%)	
Yes	164 (5.02%)	314 (7.92%)	64 (9.92%)	542 (6.88%)	

*Note*: BMI, body mass index; HbA1c, glycated hemoglobin; LDL‐C, low‐density lipoprotein cholesterol; TyG index, triglyceride‐glucose index.

### 3.2 Distribution of Depression and Frailty

**FIGURE 1:**
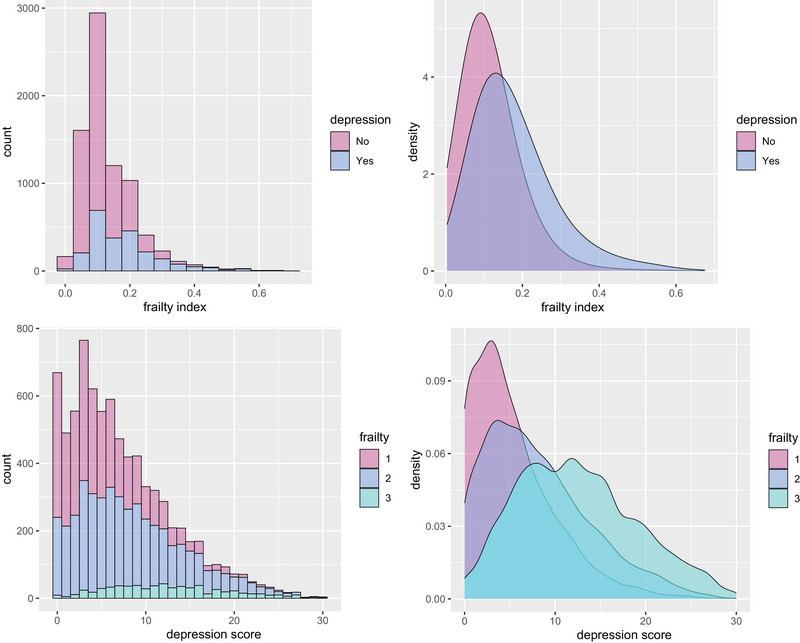
Distribution map of depression and frailty.

The distribution of depression and frailty revealed a co‐occurrence pattern. As the FI increased, the proportion of participants with depression rose correspondingly. Similarly, with higher depression scores, the proportion of individuals with more severe frailty levels increased (Figure 1). Figure S2 showed a nonlinear association between the depression score and the frailty (for nonlinearity, *p* < 0.001).

### 3.3 Associations of Frailty and Depression With Digestive, Gastrointestinal, and Liver Diseases Risk

During a median follow‐up of 9 years (2011–2020), 2076 participants developed new‐onset digestive system diseases, representing an incidence rate of 26.36%. Among these, 1751 cases involved gastrointestinal diseases (22.23%), and 542 cases were liver diseases (6.88%).

**FIGURE 2:**
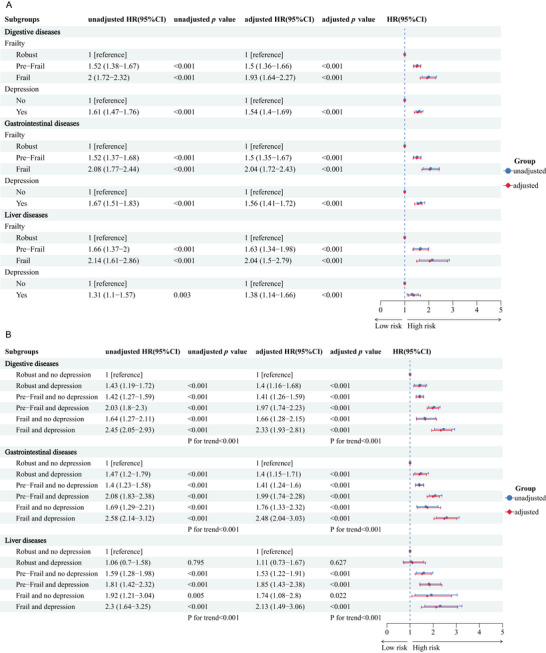
Associations of frailty and depression with digestive, gastrointestinal, and liver diseases risk. (A) Associations of frailty and depression with disease. (B) Joint associations of frailty and depression with incident disease. Adjusted model based on gender, age, LDL‐C, HbA1c, TyG index, marry status, educational level, residence, smoking status, drinking status, physical activity, BMI categorization, kidney diseases, dyslipidemia, social activity, live alone.

For digestive system diseases, individuals in the pre‐frail group had an HR of 1.5 (95% CI: 1.36–1.66) compared to those in the robust group, whereas the HR for the frail group was 1.93 (95% CI: 1.64–2.27). Similarly, depression was associated with an increased risk of digestive system diseases (HR = 1.54, 95% CI: 1.4–1.69). These associations were consistent for gastrointestinal (frailty HR = 2.04, 95% CI: 1.72–2.43; depression HR = 1.56, 95% CI: 1.41–1.72) and liver diseases (frailty HR = 2.04, 95% CI: 1.5–2.79; depression HR = 1.38, 95% CI: 1.14–1.66) after adjusting for potential confounders (Figure 2A).

The combined impact of frailty and depression on the risk of digestive, gastrointestinal, and liver diseases was examined. Compared to participants who were robust and without depression, those who were frail and depressed had significantly highest risks of digestive diseases (HR = 2.33, 95% CI: 1.93–2.81), gastrointestinal diseases (HR = 2.48, 95% CI: 2.04–3.03), and liver diseases (HR = 2.13, 95% CI: 1.49–3.06) after adjusting for potential confounders (Figure 2B).

**FIGURE 3:**
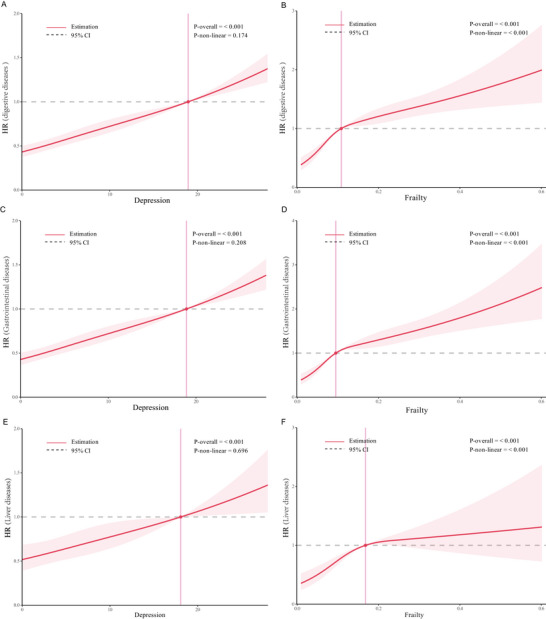
RCS curves of frailty index and depression score with digestive, gastrointestinal, and liver diseases. (A) RCS curves of depression score with digestive diseases. (B) RCS curves of frailty index with digestive diseases. (C) RCS curves of depression score with gastrointestinal diseases. (D) RCS curves of frailty index with gastrointestinal diseases. (E) RCS curves of depression score with liver diseases. (F) RCS curves of frailty index with liver diseases. All model adjusted for gender, age, LDL‐C, HbA1c, TyG index, marry status, educational level, residence, smoking status, drinking status, physical activity, BMI categorization, kidney diseases, dyslipidemia, social activity, live alone.

RCS regression analysis revealed a linear association between depression score and the risk of digestive system diseases (nonlinearity *p* = 0.174). In contrast, FI demonstrated a significant nonlinear positive association with digestive disease risk (nonlinearity *p* < 0.001) (Figure 3A–F).

### 3.4 Joint and Interactive Effects and Mutual Mediation Effects of Frailty and Depression on Disease Risk

No significant additive interactions were observed between frailty and depression for any of the disease outcomes (Table S3). However, an analysis of the overall risk of digestive and gastrointestinal diseases revealed a multiplicative interaction between depression and frailty (Table S3).

**FIGURE 4:**
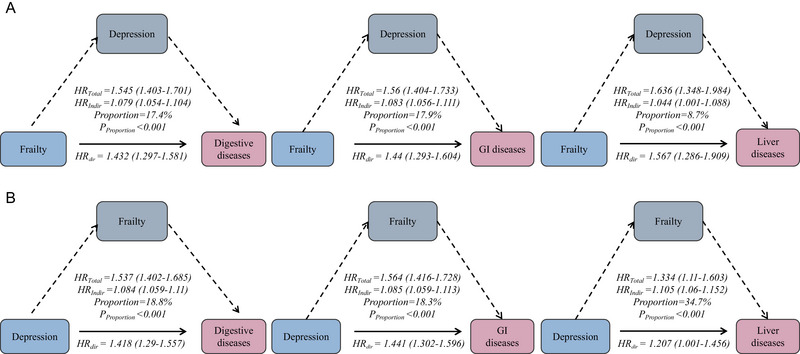
Mutual mediation effects of the frailty and depression on digestive, gastrointestinal, and liver diseases. (A) Mutual mediation effects of the depression on digestive, gastrointestinal, and liver diseases. (B) Mutual mediation effects of the frailty on digestive, gastrointestinal, and liver diseases. All model adjusted for gender, age, LDL‐C, HbA1c, TyG index, marry status, educational level, residence, smoking status, drinking status, physical activity, BMI categorization, kidney diseases, dyslipidemia, social activity, live alone.

Mediation analysis demonstrated that depression mediated part of the association between frailty and disease risk. For digestive, gastrointestinal, and liver diseases, the proportions mediated by depression were 17.40%, 17.9%, and 8.7%, respectively (Figure 4). Conversely, frailty mediated 18.8% of the relationship between depression and digestive diseases, 18.3% for gastrointestinal diseases, and 34.7% for liver diseases.

### 3.5 Predictive Value of Frailty and Depression

**FIGURE 5:**
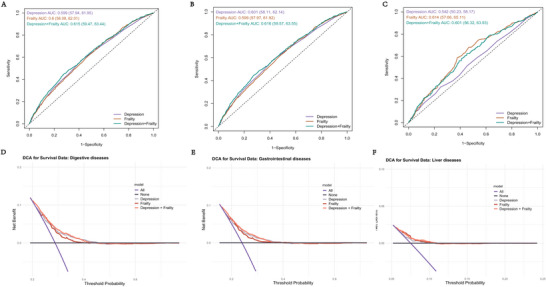
ROC curve and DCA curve of frailty and depression for predicting diseases. (A–C) The receiver operating characteristic (ROC) curve evaluating the discriminative capabilities by calculating the AUC; (D–F) decision curve analysis to compare the clinical utility, the *y*‐axis represents net benefits, calculated by subtracting the relative harm (false positives) from the benefits (true positives). AUC, area under the curve; DCA, decision curve analysis.

The combined frailty and depression index improved the predictive ability for all disease outcomes. The area under the curve (AUC) for digestive, gastrointestinal, and liver diseases was 0.600, 0.599, and 0.614, respectively (Figure 5A–C). DCA confirmed the clinical utility of the combined index (Figure 5D–F). Notably, NRI and IDI indicated significantly enhanced predictive performance for the combined index compared to individual frailty or depression scores, except for liver diseases (Table S4). These findings underscore the superior predictive efficacy of the combined index.

### 3.6 Associations of Joint Trajectories on Frailty and Depression With Diseases Risk

**FIGURE 6:**
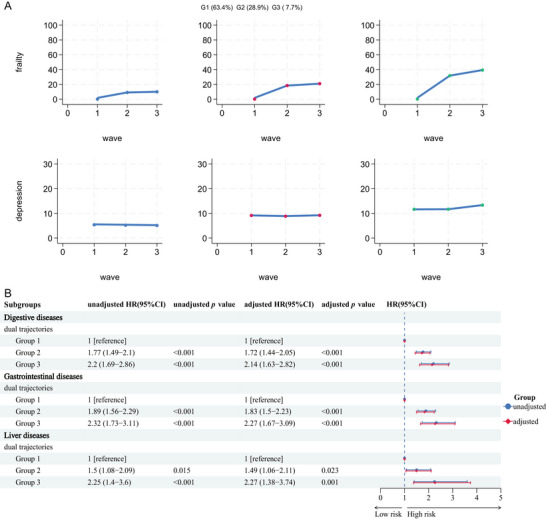
Associations of joint trajectories on frailty and depression with digestive, gastrointestinal, and liver diseases. (A) The combined change trajectory of the weakness index and the depression score over the waves. (B) Association between each trajectory group and the risk of the disease. Adjusted model based on gender, age, LDL‐C, HbA1c, TyG index, marry status, educational level, residence, smoking status, drinking status, physical activity, BMI categorization, kidney diseases, dyslipidemia, social activity, live alone.

A trajectory model was developed using FI values and depression scores measured at waves 1–3 to categorize the population into three distinct groups. Group 1 (stable group) comprised 63.4% of the population and was characterized by minimal fluctuations in both the FI and depression scores over time. Group 2 (moderately worsening group) included 28.9% of the population and was defined by consistently high‐to‐medium depression scores alongside a gradual increase in the FI. Group 3 (significant worsening group) represented 7.7% of the population and was marked by persistently high, slightly increasing depression scores and a pronounced rise in the FI, indicating substantial change. After adjusting for covariates, individuals in Group 2 exhibited a 72% higher risk of digestive diseases, whereas those in Group 3 experienced a 114% greater risk compared to Group 1 (Figure 6). Similarly, the relative risks for gastrointestinal system and liver diseases were notably elevated in both Groups 2 and 3 compared to Group 1 (Figure 6).

### 3.7 Sensitivity Analysis

In the stratified analyses, the findings were largely consistent across both males and females, as well as between middle‐aged and older participants. Similarly, in repeated analyses conducted on the dataset without interpolation, the majority of results remained stable. Details of the five duplicate analyses are presented in Figures S3–S12 and Tables S5–S13. The inclusion of the FI and depression score significantly enhanced the AUC of the traditional model for total digestive disorders, demonstrating improved discrimination and risk reclassification, as indicated by an IDI of 0.02 (95% CI 0.003–0.025; *p* < 0.001) and an NRI of 0.141 (0.025, 0.167; *p* < 0.001). These findings suggest the superior efficacy of the composite index in this study. Comparable results were observed in the subtypes of liver and gastrointestinal diseases (Figure S13 and Table S14). The *E* values indicated that the associations of depression and frailty with all diseases were robust (Table S15).

## 4 Discussion

This large‐scale prospective cohort study seeks to explore the mediating, interactive, and joint impacts of two critical factors—frailty and depression—on gastrointestinal disease risk. The results provide new perspectives on the complex relationships between these variables and their role in assessing gastrointestinal risks, delivering important implications for healthcare practices and public health policies.

Our study highlights the well‐recognized association between frailty and increased risk of digestive diseases, corroborating findings from previous research (Bellelli et al. 2024; He et al. 2024). Notably, frailty is associated with systemic inflammation, immunosenescence, and gut microbiota dysbiosis, which collectively contribute to digestive tract dysfunction. Chronic low‐grade inflammation, a hallmark of frailty, is linked to elevated levels of pro‐inflammatory cytokines such as IL‐6 and TNF‐α, which impair mucosal integrity and increase susceptibility to infections and inflammatory digestive diseases (Di Sabatino et al. 2018). Frailty is also commonly associated with nutritional deficiencies, which impair the repair and maintenance of digestive tissue and exacerbate disease progression (Pu et al. 2024). In addition, individuals with frailty are more likely to experience reduced gastrointestinal motility, leading to complications such as constipation, small intestinal bacterial overgrowth (SIBO), and an increased risk of diverticular disease (Patel and Winer 2024). Emerging evidence suggests that dysbiosis of the gut microbiota—a reduction in diversity and an imbalance between harmful and beneficial bacteria—is a critical mediator of frailty's impact on digestive health. Dysbiosis has been shown to promote systemic inflammation and increase intestinal permeability, further compounding disease risk (Patel and Winer 2024). Therefore, chronic inflammation, impaired nutrient absorption, and gut microbiota imbalance may represent key mechanisms underlying the association between frailty and digestive disease risk.

Our study highlights the well‐documented association between depression and an increased risk of digestive diseases, supported by several underlying mechanisms. Depression disrupts the gut–brain axis, a bidirectional communication network between the central nervous system and the digestive tract, leading to altered motility and sensitivity, commonly seen in conditions like irritable bowel syndrome (IBS) (Chen et al. 2023; Wang et al. 2021). Additionally, depression is associated with dysbiosis—imbalances in gut microbiota composition—that increase intestinal permeability and trigger systemic inflammation, further contributing to digestive disorders (Chen et al. 2022; Gonçalves et al. 2024). Chronic inflammation, characterized by elevated levels of pro‐inflammatory cytokines such as IL‐6 and TNF‐α in individuals with depression, impairs digestive mucosal integrity, heightening susceptibility to infections and inflammatory diseases (Truyens et al. 2022). Moreover, autonomic nervous system dysfunction, often linked to depression, disrupts digestive motility and secretion, resulting in symptoms such as constipation and diarrhea (Bajkó et al. 2012). Depression also activates the hypothalamic–pituitary–adrenal (HPA) axis, increasing cortisol levels and stress hormone production, which adversely affect digestive function and promote conditions like peptic ulcers and gastroesophageal reflux disease (GERD) (Chen et al. 2023; Mayer 2000). These mechanisms underscore the critical role of mental health management in mitigating the risk and progression of digestive diseases.

Our study further explored the joint and interactive effects of frailty and depression on the risk of digestive diseases. Contrary to our initial expectations, the findings did not reveal a significant additive interaction between these two factors in increasing digestive disease risk. However, the significant interaction effect under the multiplicative model revealed a synergistic effect between the two in jointly influencing the risk of gastrointestinal diseases. Although both frailty and depression independently contribute to digestive disorders through mechanisms such as chronic inflammation, altered gut motility, and microbiota dysbiosis (Njei et al. 2024; DeJong et al. 2020; Liu et al. 2024; Rehman 2012; Fan et al. 2024; Salwen‐Deremer et al. 2024), their combined impact does not appear to exceed the sum of their individual effects. This suggests that the interplay between frailty and depression in the context of digestive diseases may be more complex than previously understood.

Digestive diseases are multifactorial conditions influenced by a wide array of interwoven factors, including diet, medication use, immune status, and autonomic nervous system regulation (Sun et al. 2024; Almeida et al. 2022; Wang et al. 2024; Massironi et al. 2024; Effinger et al. 2019; Suri et al. 2024; Woodie et al. 2024). These variables may moderate the interaction between frailty and depression, potentially explaining the lack of a pronounced synergistic effect. Another possibility is that frailty and depression operate through partially overlapping but distinct biological pathways. For instance, although depression may disrupt the gut–brain axis, frailty could exacerbate physical vulnerability through malnutrition and immune dysfunction. Further research is needed to elucidate how these pathways interact in contributing to digestive disease risk.

Although a synergistic interaction was not observed, the joint assessment of frailty and depression provides valuable insights for risk stratification. Individuals exhibiting both frailty and depression demonstrated a higher cumulative risk of digestive diseases compared to those affected by either factor alone. Notably, a stronger association with severe digestive complications was observed in individuals with advanced frailty combined with depression, suggesting a potential threshold effect. This finding aligns with the hypothesis that disease risk may not increase linearly with exposure but may intensify beyond certain critical thresholds.

Our findings indicate a significant mutual mediating effect between frailty and depression in the context of digestive diseases. Previous studies have highlighted that depression can exacerbate gastrointestinal dysfunction through pathways such as altered gut–brain axis communication and increased pro‐inflammatory cytokine levels, which are also hallmark features of frailty (Yuan et al. 2023). Similarly, frailty has been shown to increase vulnerability to digestive diseases by promoting immune dysfunction, chronic inflammation, and malnutrition, all of which can worsen depressive symptoms (Xu et al. 2022). Inflammation may be a key mediator between frailty and depression (Carbery et al. 2024; Marcos‐Pérez et al. 2020), and pro‐inflammatory cytokines such as IL‐6 and TNF‐α may promote the mediating effect between frailty and depression (Di Sabatino et al. 2018; Truyens et al. 2022; Harneshaug et al. 2019).

This mutual mediation underscores the complex interplay between frailty and depression in driving digestive disease risk. Our study highlights the joint contributions of frailty and depression as determinants for digestive health outcomes. These findings underscore the importance of integrated care approaches that address both physical and mental health to mitigate the risk of digestive diseases. Further research is needed to elucidate the specific biological pathways underpinning this interaction.

This article investigates the relationship between the joint trajectories of vulnerability and depression and their associated disease risk. Using vulnerability index values and depression scores collected over waves 1–3, we categorized the study population into three groups: Group 1 (stable group), Group 2 (moderately worsening group), and Group 3 (significant worsening group). The study revealed that individuals in Groups 2 and 3 exhibited a significantly higher risk of developing digestive, gastrointestinal, and liver disorders compared to those in Group 1. These findings suggest that as vulnerability and depression progressively worsen, the risk of developing digestive system diseases increases markedly. This observation aligns with existing literature that underscores the role of vulnerability and depression in the development of chronic diseases. Moreover, it highlights the importance of monitoring changes in vulnerability and depression as potential early indicators of an individual's susceptibility to gastrointestinal and hepatic diseases. Consequently, interventions aimed at mitigating these changes—particularly those targeting vulnerability and depressive symptoms—may help reduce the risk of associated diseases.

These findings have practical implications for clinical practice and public health strategies. Early identification of individuals at high risk based on combined frailty and depression assessments allows for more targeted interventions. Comprehensive care strategies emphasizing mental health support, nutritional optimization, and regular monitoring of digestive health should be prioritized for this population. Furthermore, tailored therapeutic approaches addressing both physical and psychological vulnerability may help mitigate the compounded risk of digestive diseases associated with frailty and depression.

### 4.1 Strengths and Limitations

This study has several strengths. First, it utilized data from the CHARLS, a large, nationally representative cohort, which enhances the generalizability of the findings to middle‐aged and older adults in China. The 9‐year follow‐up allowed for a long‐term assessment of the impact of frailty and depression on the development of digestive diseases. Additionally, the study employed robust statistical techniques, such as Cox regression and mediation models, to explore both the independent and combined effects of frailty and depression. The use of a combined frailty and depression index showed improved predictive accuracy for digestive diseases, suggesting potential clinical utility for risk stratification.

However, there are several limitations. Despite adjusting for several confounders, factors such as dietary habits, physical activity, and genetic predispositions may have introduced residual confounding. The study was also limited to a Chinese adult population, which may reduce the generalizability to other ethnic groups. As an observational study, it cannot establish causality, and further randomized controlled trials are needed to confirm these associations. Lastly, the reliance on self‐reported data for frailty and depression may introduce bias, and future studies should incorporate more objective measures or clinical diagnoses to improve accuracy.

## 5 Conclusions

The findings underscore the combined impact and mutual mediation of frailty and depression in the development of digestive disorders, with progressively increasing FI and persistent high depression levels being associated with the highest digestive diseases risk. Thus, integrating the assessment of frailty and depression should be prioritized in residual risk stratification and primary prevention strategies for digestive diseases.

## Author Contributions

Xiaofei Fan and Xiaoming Qiao collected relevant data and drafted manuscripts. Tao Han and Qianqian Wu reviewed and made significant revisions to the manuscript. Tao Han guided the preparation of this manuscript. All authors have read and approved the final manuscript.

## Ethics Statement

This study is a secondary analysis of publicly available data from the de‐identified China Health and Aging Longitudinal Study (CHARLS). The original CHARLS study was approved by the Biomedical Ethics Review Board of Peking University (IRB00001052‐11015), and written informed consent was obtained from all participants. All methods were conducted in accordance with the principles outlined in the declaration of Helsinki.

## Consent

The authors have nothing to report.

## Conflicts of Interest

The authors declare no conflicts of interest.

## Peer Review

The peer review history for this article is available at https://publons.com/publon/10.1002/brb3.70877

## Supporting information




**Supplementary Materials**: brb370877‐sup‐0001‐SuppMatt.docx

## Data Availability

Some or all data generated or analyzed during this study are included in this published article or in the data repositories listed in “References.”
