# Supplementary material for: Association Between the Combined Effects and Joint Trajectories of Depression and Frailty and the Risk of Digestive Diseases: A Longitudinal Study
Source: Brain Behav. 2025 Sep 21;15(9):e70877. doi: 10.1002/brb3.70877 (PMC12451021; doi:10.1002/brb3.70877)
Supplement: Supplementary file 1 — Supplementary Materials: brb370877‐sup‐0001‐SuppMatt.docx [file BRB3-15-e70877-s001.docx]

**Figure S1.** Flowchart of the study population.

**
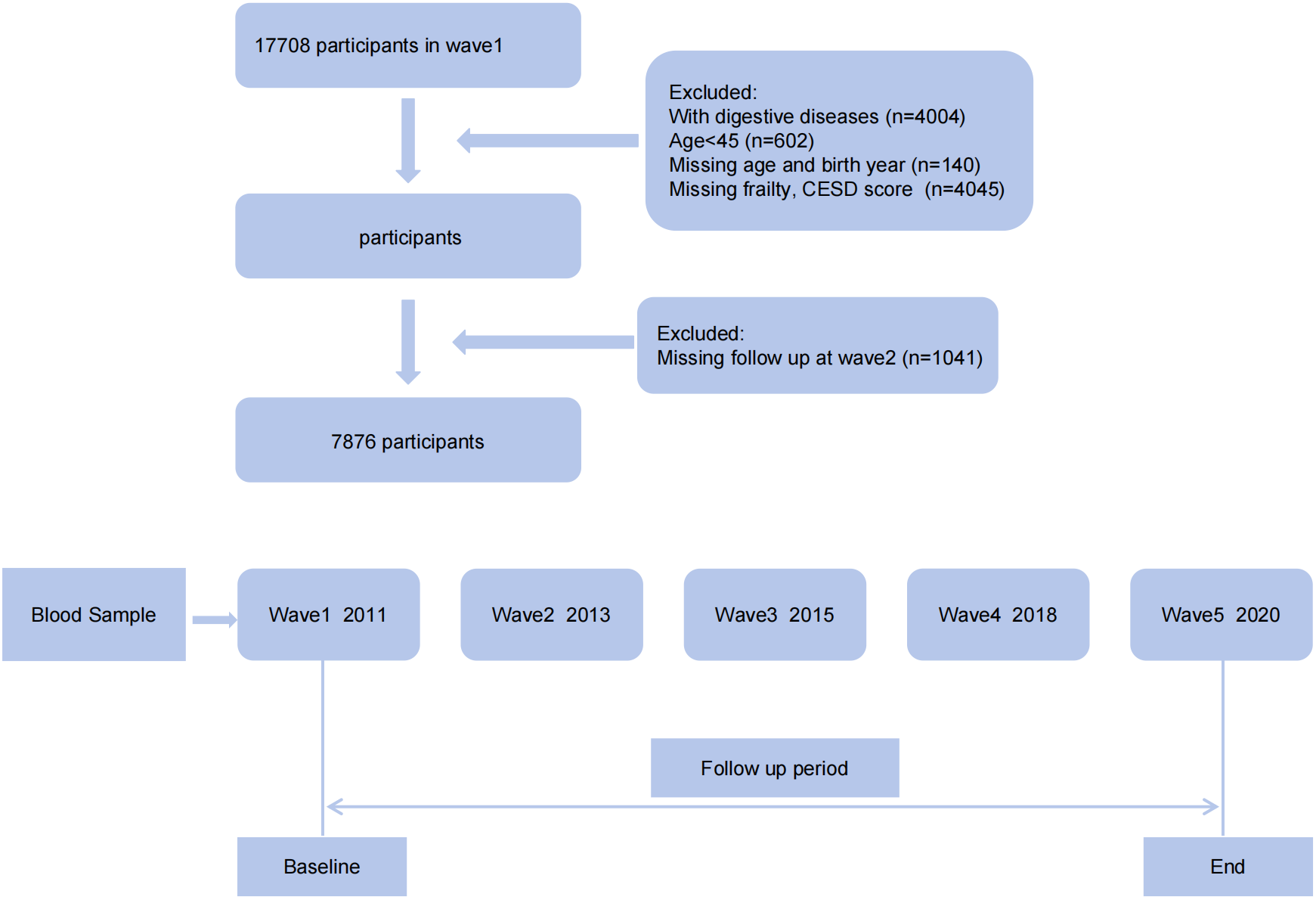
**

**Table S1.** The 31 items used to construct the frailty index.

| No | Description of the items | Cut-off value |
| --- | --- | --- |
|  | CHARLS |  |
| 1 | Self-reported physician diagnosed hypertension | Yes = 1, No = 0 |
| 2 | Self-reported physician diagnosed diabetes | Yes = 1, No = 0 |
| 3 | Self-reported physician diagnosed heart disease | Yes = 1, No = 0 |
| 4 | Self-reported physician diagnosed stroke | Yes = 1, No = 0 |
| 5 | Self-reported physician diagnosed cancer | Yes = 1, No = 0 |
| 6 | Self-reported physician diagnosed arthritis | Yes = 1, No = 0 |
| 7 | Self-reported physician diagnosed chronic lung disease | Yes = 1, No = 0 |
| 8 | Self-reported physician diagnosed asthma | Yes = 1, No = 0 |
| 9 | Self-reported physician diagnosed any emotional, nervous, or psychiatric problems | Yes = 1, No = 0 |
| 10 | Self-reported physician diagnosed memory-related disease | Yes = 1, No = 0 |
| 11 | Self-reported vision problems | Yes = 1, No = 0 in the CHARLS; Poor or fair = 1, excellent, very good, or good = 0 in the ELSA |
| 12 | Self-reported hearing problems | Yes = 1, No = 0 in the CHARLS;  Poor or fair = 1, excellent, very good, or good = 0 in the ELSA |
| 13 | Self-reported general health status | Poor or fair = 1, excellent, very good, or good = 0 |
| 14 | Difficulty with dressing | Yes = 1, No = 0 |
| 15 | Difficulty with bathing or showering | Yes = 1, No = 0 |
| 16 | Difficulty with eating | Yes = 1, No = 0 |
| 17 | Difficulty with getting in and out of bed | Yes = 1, No = 0 |
| 18 | Difficulty with using the toilet | Yes = 1, No = 0 |
| 19 | Difficulty with managing money | Yes = 1, No = 0 |
| 20 | Difficulty with taking medications | Yes = 1, No = 0 |
| 21 | Difficulty with shopping for groceries | Yes = 1, No = 0 |
| 22 | Difficulty with preparing meals | Yes = 1, No = 0 |
| 23 | Difficulty with doing housework | Yes = 1, No = 0 |
| 24 | Mobility: difficulty with walking 100 yards | Yes = 1, No = 0 |
| 25 | Mobility: difficulty with getting up from a chair after sitting for long periods | Yes = 1, No = 0 |
| 26 | Mobility: difficulty with climbing several flights of stairs without resting | Yes = 1, No = 0 |
| 27 | Mobility: difficulty with lifting or carrying weights over 5 kg | Yes = 1, No = 0 |
| 28 | Mobility: difficulty with picking up a coin from the table | Yes = 1, No = 0 |
| 29 | Mobility: difficulty with stooping, kneeling, or crouching | Yes = 1, No = 0 |
| 30 | Mobility: difficulty with reaching arms above shoulder level | Yes = 1, No = 0 |
| 31 | Cognition: (memory test score + orientation test score) **/** 14 | Continuous, ranging from 0 to 1 |

Heart disease indicates the angina, coronary heart disease, congestive heart failure, or other heart problems.

Memory-related disease indicates Alzheimer’s disease or dementia, organic brain senility, or other serious memory impairment. The memory score is the average of words that are not recalled in the immediate and delayed word recall tasks. The memory score ranges from 0 to 10. The orientation test comprises four questions about the day of the week, the month, the date of the month, and the year. One point is given for each wrong answer, and the range is from 0 to 4.

**Table S2.** Baseline characteristics of participants from different depression groups.

| ***Groups*** | ***No depression*** | ***Depression*** | ***Overall*** | ***p*** |
| --- | --- | --- | --- | --- |
|  | ***(N=5558)*** | ***(N=2318)*** | ***(N=7876)*** |  |
| **Gender, n(%)** |  |  |  | <0.001 |
| male | 3057 (55.00%) | 974 (42.02%) | 4031 (51.18%) |  |
| female | 2501 (45.00%) | 1344 (57.98%) | 3845 (48.82%) |  |
| **Age** | 57.00 [50.00;64.00] | 58.00 [52.00;64.00] | 57.00 [51.00;64.00] | <0.001 |
| **Frailty index** | 0.10 [0.07;0.15] | 0.15 [0.11;0.22] | 0.11 [0.08;0.17] | <0.001 |
| **Frailty** |  |  |  | <0.001 |
| Robust | 2727 (49.06%) | 538 (23.21%) | 3265 (41.46%) |  |
| Pre-Frail | 2591 (46.62%) | 1375 (59.32%) | 3966 (50.36%) |  |
| Frail | 240 (4.32%) | 405 (17.47%) | 645 (8.19%) |  |
| **HbA1c (%)** | 5.10 [4.80;5.50] | 5.11 [4.86;5.50] | 5.10 [4.80;5.50] | 0.142 |
| **LDL-C (mg/dl)** | 115.21 [93.94;137.63] | 113.66 [92.78;136.86] | 114.82 [93.56;137.24] | 0.304 |
| **TyG index** | 8.66 [8.24;9.11] | 8.64 [8.23;9.09] | 8.65 [8.23;9.10] | 0.602 |
| **Marry status** |  |  |  | <0.001 |
| Other | 502 (9.03%) | 348 (15.01%) | 850 (10.79%) |  |
| Married | 5056 (90.97%) | 1970 (84.99%) | 7026 (89.21%) |  |
| **Educational level** |  |  |  | <0.001 |
| Less than upper secondary education | 4628 (83.27%) | 2127 (91.76%) | 6755 (85.77%) |  |
| Upper secondary | 784 (14.11%) | 168 (7.25%) | 952 (12.09%) |  |
| Tertiary education | 146 (2.63%) | 23 (0.99%) | 169 (2.15%) |  |
| **Residence** |  |  |  | <0.001 |
| Urban | 2462 (44.30%) | 772 (33.30%) | 3234 (41.06%) |  |
| Rural | 3096 (55.70%) | 1546 (66.70%) | 4642 (58.94%) |  |
| **Smoking status** |  |  |  | <0.001 |
| No | 3699 (66.55%) | 1625 (70.10%) | 5324 (67.60%) |  |
| Yes | 1859 (33.45%) | 693 (29.90%) | 2552 (32.40%) |  |
| **Drinking status** |  |  |  | <0.001 |
| No | 3427 (61.66%) | 1611 (69.50%) | 5038 (63.97%) |  |
| Yes | 2131 (38.34%) | 707 (30.50%) | 2838 (36.03%) |  |
| **Physical activity** |  |  |  | 0.528 |
| No | 583 (10.49%) | 255 (11.00%) | 838 (10.64%) |  |
| Yes | 4975 (89.51%) | 2063 (89.00%) | 7038 (89.36%) |  |
| **BMI categorization** |  |  |  | <0.001 |
| underweight less than 18.5 | 549 (9.88%) | 276 (11.91%) | 825 (10.47%) |  |
| normal weight from 18.5 to 23.9 | 1922 (34.58%) | 937 (40.42%) | 2859 (36.30%) |  |
| overweight from 23 to 24.9 | 1051 (18.91%) | 395 (17.04%) | 1446 (18.36%) |  |
| obesity from 25 to 100 | 2036 (36.63%) | 710 (30.63%) | 2746 (34.87%) |  |
| **Chronic kidney diseases** |  |  |  | <0.001 |
| No | 5380 (96.80%) | 2186 (94.31%) | 7566 (96.06%) |  |
| Yes | 178 (3.20%) | 132 (5.69%) | 310 (3.94%) |  |
| **Dyslipidemia** |  |  |  | 0.900 |
| No | 5042 (90.72%) | 2100 (90.60%) | 7142 (90.68%) |  |
| Yes | 516 (9.28%) | 218 (9.40%) | 734 (9.32%) |  |
| **Social activity** |  |  |  | <0.001 |
| No | 2942 (52.93%) | 998 (43.05%) | 3940 (50.03%) |  |
| Yes | 2616 (47.07%) | 1320 (56.95%) | 3936 (49.97%) |  |
| **Live alone** |  |  |  | 0.004 |
| No | 5308 (95.50%) | 2172 (93.70%) | 7480 (94.97%) |  |
| Yes | 250 (4.50%) | 146 (6.30%) | 396 (5.03%) |  |
| **Incident digestive diseases** |  |  |  | <0.001 |
| No | 4273 (76.88%) | 1527 (65.88%) | 5800 (73.64%) |  |
| Yes | 1285 (23.12%) | 791 (34.12%) | 2076 (26.36%) |  |
| **Incident gastrointestinal diseases** |  |  |  | <0.001 |
| No | 4494 (80.86%) | 1631 (70.36%) | 6125 (77.77%) |  |
| Yes | 1064 (19.14%) | 687 (29.64%) | 1751 (22.23%) |  |
| **Incident liver diseases** |  |  |  | <0.001 |
| No | 5206 (93.67%) | 2128 (91.80%) | 7334 (93.12%) |  |
| Yes | 352 (6.33%) | 190 (8.20%) | 542 (6.88%) |  |

note: HbA1c: Glycated Hemoglobin; LDL-C: Low Density Lipoprotein Cholesterol; TyG index: triglyceride-glucose index.

**Table S3.** Interactive effects of frailty and depression on incident digestive, gastrointestinal, and liver diseases.

| ***Interactive items (frailty and loneliness)*** | ***Digestive diseases*** | | ***Gastrointestinal diseases*** | | ***Liver diseases*** | |
| --- | --- | --- | --- | --- | --- | --- |
|  | ***unadjusted model*** | ***adjusted model*** | ***unadjusted model*** | ***adjusted model*** | ***unadjusted model*** | ***adjusted model*** |
| **Additive effects** |  |  |  |  |  |  |
| RERI | 0.005 (-0.009, 0.019) | -0.007 (-0.023, 0.009) | 0.006 (-0.009, 0.021) | -0.01 (-0.027, 0.008) | 0.015 (-0.019, 0.049) | 0.009 (-0.026, 0.044) |
| AP | 0.003 (-0.006, 0.012) | -0.005 (-0.016, 0.006) | 0.004 (-0.006, 0.013) | -0.007 (-0.019, 0.005) | 0.009 (-0.014, 0.032) | 0.006 (-0.018, 0.031) |
| SI | 1.009 (0.982, 1.037) | 0.985 (0.953, 1.018) | 1.01 (0.981, 1.04) | 0.977 (0.942, 1.014) | 1.025 (0.954, 1.102) | 1.019 (0.937, 1.109) |
| **Multiplicative effect** |  |  |  |  |  |  |
| INTM | 0.987 (0.971, 1.004) | **0.982 (0.966, 0.999)** | 0.987 (0.97, 1.005) | **0.981 (0.963, 0.999)** | 1.004 (0.969, 1.04) | 1 (0.965, 1.036) |

**Table S4.** NDI and IDI index for frailty and depression combinations.

| **Comparison** | **IDI (95% CI)** | ***P*_NRI_** | **NRI (95% CI)** | ***P*_IDI_** |
| --- | --- | --- | --- | --- |
| **Digestive diseases** |  |  |  |  |
| Depression vs. Frailty | -0.002(-0.01, 0.001) | 0.727 | -0.037(-0.103, 0.007) | 0.182 |
| Depression vs. Frailty + Depression | 0.006(0.001, 0.01) | **<0.001** | 0.089(0.014, 0.128) | **<0.001** |
| Frailty vs. Frailty + Depression | 0.008(0.001, 0.011) | **<0.001** | 0.109(0.018, 0.138) | **<0.001** |
| **Gastrointestinal diseases** |  |  |  |  |
| Depression vs. Frailty | -0.003(-0.007, 0.001) | 0.364 | -0.038(-0.082, -0.001) | **<0.001** |
| Depression vs. Frailty + Depression | 0.006(0.001, 0.01) | **<0.001** | 0.086(0.014, 0.115) | **<0.001** |
| Frailty vs. Frailty + Depression | 0.009(0.001, 0.011) | **<0.001** | 0.112(0.018, 0.147) | **<0.001** |
| **Liver diseases** |  |  |  |  |
| Depression vs. Frailty | 0.003(0, 0.005) | **<0.001** | 0.104(0.011, 0.203) | **<0.001** |
| Depression vs. Frailty + Depression | 0.003(0, 0.005) | **<0.001** | 0.148(0.02, 0.209) | **<0.001** |
| Frailty vs. Frailty + Depression | 0(0, 0.001) | 0.727 | 0.022(-0.044, 0.058) | 0.364 |

**Figure S2.** Linear association between depression score and frailty index.

****Graphs show β for depression score adjusted for gender, age, LDL-C, HbA1c, TyG index, marry status, educational level, residence, smoking status, drinking status, physical activity, BMI categorization, kidney diseases, dyslipidemia, social activity, live alone. Data were fitted by a restricted cubic spline (RCS) linear regression model. Solid lines indicate β, and shadow shapes indicate 95% CIs.

**Figure S3.** Associations of depression and frailty with disease in males.

Adjusted model based on gender, age, LDL-C, HbA1c, TyG index, marry status, educational level, residence, smoking status, drinking status, physical activity, BMI categorization, kidney diseases, dyslipidemia, social activity, live alone.

**Figure S4.** Joint associations of depression and frailty combinations with incident diseases in males.

Adjusted model based on gender, age, LDL-C, HbA1c, TyG index, marry status, educational level, residence, smoking status, drinking status, physical activity, BMI categorization, kidney diseases, dyslipidemia, social activity, live alone.

**Figure S5.** Associations of depression and frailty with disease in females.

Adjusted model based on gender, age, LDL-C, HbA1c, TyG index, marry status, educational level, residence, smoking status, drinking status, physical activity, BMI categorization, kidney diseases, dyslipidemia, social activity, live alone.

**Figure S6.** Joint associations of depression and frailty combinations with incident diseases in females.

Adjusted model based on gender, age, LDL-C, HbA1c, TyG index, marry status, educational level, residence, smoking status, drinking status, physical activity, BMI categorization, kidney diseases, dyslipidemia, social activity, live alone.

**Figure S7.** Associations of depression and frailty with disease in individuals aged under 60.

Adjusted model based on gender, age, LDL-C, HbA1c, TyG index, marry status, educational level, residence, smoking status, drinking status, physical activity, BMI categorization, kidney diseases, dyslipidemia, social activity, live alone.

**Figure S8.** Joint associations of depression and frailty combinations with incident diseases in individuals aged under 60.

Adjusted model based on gender, age, LDL-C, HbA1c, TyG index, marry status, educational level, residence, smoking status, drinking status, physical activity, BMI categorization, kidney diseases, dyslipidemia, social activity, live alone.

**Figure S9.** Associations of depression and frailty with disease in individuals aged over 60.

Adjusted model based on gender, age, LDL-C, HbA1c, TyG index, marry status, educational level, residence, smoking status, drinking status, physical activity, BMI categorization, kidney diseases, dyslipidemia, social activity, live alone.

**Figure S10.** Joint associations of depression and frailty combinations with incident diseases in individuals aged over 60.

Adjusted model based on gender, age, LDL-C, HbA1c, TyG index, marry status, educational level, residence, smoking status, drinking status, physical activity, BMI categorization, kidney diseases, dyslipidemia, social activity, live alone.

**Figure S11.** Associations of depression and frailty with disease in the non-imputed dataset.

Adjusted model based on gender, age, LDL-C, HbA1c, TyG index, marry status, educational level, residence, smoking status, drinking status, physical activity, BMI categorization, kidney diseases, dyslipidemia, social activity, live alone.

**Figure S12.** Joint associations of depression and frailty combinations with incident diseases in the non-imputed dataset.

Adjusted model based on gender, age, LDL-C, HbA1c, TyG index, marry status, educational level, residence, smoking status, drinking status, physical activity, BMI categorization, kidney diseases, dyslipidemia, social activity, live alone.

Table S5. Interactive effects of frailty and depression on incident disease stratified by gender.

| **Group** |  | **Term** | **Interactive effect (95% CI)** |
| --- | --- | --- | --- |
| **Digestive diseases** |  |  |  |
|  | **Female** | RERI | -0.017 (-0.038, 0.004) |
|  |  | AP | -0.012 (-0.025, 0.002) |
|  |  | SI | 0.966 (0.929, 1.004) |
|  |  | INTM | **0.971 (0.949, 0.993)** |
|  | **Male** | RERI | 0.005 (-0.02, 0.031) |
|  |  | AP | 0.004 (-0.014, 0.021) |
|  |  | SI | 1.011 (0.954, 1.072) |
|  |  | INTM | 0.995 (0.97, 1.021) |
| **Gastrointestinal diseases** |  |  |  |
|  | **Female** | RERI | -0.024 (-0.046, -0.001) |
|  |  | AP | -0.016 (-0.03, -0.002) |
|  |  | SI | 0.953 (0.913, 0.995) |
|  |  | INTM | **0.967 (0.944, 0.99)** |
|  | **Male** | RERI | 0.011 (-0.016, 0.038) |
|  |  | AP | 0.008 (-0.013, 0.028) |
|  |  | SI | 1.029 (0.944, 1.122) |
|  |  | INTM | 1.001 (0.972, 1.03) |
| **Liver diseases** |  |  |  |
|  | **Female** | RERI | 0.022 (-0.023, 0.067) |
|  |  | AP | 0.017 (-0.022, 0.057) |
|  |  | SI | 1.083 (0.801, 1.465) |
|  |  | INTM | 1.013 (0.959, 1.07) |
|  | **Male** | RERI | -0.016 (-0.079, 0.047) |
|  |  | AP | -0.008 (-0.04, 0.023) |
|  |  | SI | 0.983 (0.924, 1.046) |
|  |  | INTM | 0.981 (0.935, 1.029) |

Adjusted model based on gender, age, LDL-C, HbA1c, TyG index, marry status, educational level, residence, smoking status, drinking status, physical activity, BMI categorization, kidney diseases, dyslipidemia, social activity, live alone. RERI, relative excess risk due to interaction; AP, proportion attributable to interaction; SI, synergy index; INTM, interaction term.

Table S6. Interactive effects of frailty and depression on incident disease stratified by age.

| **Group** |  | **Term** | **Interactive effect (95% CI)** |
| --- | --- | --- | --- |
| **Digestive diseases** |  |  |  |
|  | **age<60** | RERI | -0.001 (-0.02, 0.018) |
|  |  | AP | -0.001 (-0.014, 0.012) |
|  |  | SI | 0.998 (0.956, 1.041) |
|  |  | INTM | 0.986 (0.966, 1.006) |
|  | **age≥60** | RERI | -0.013 (-0.042, 0.016) |
|  |  | AP | -0.009 (-0.029, 0.011) |
|  |  | SI | 0.967 (0.907, 1.031) |
|  |  | INTM | 0.981 (0.952, 1.011) |
| **Gastrointestinal diseases** |  |  |  |
|  | **age<60** | RERI | -0.001 (-0.021, 0.019) |
|  |  | AP | -0.001 (-0.015, 0.014) |
|  |  | SI | 0.998 (0.948, 1.05) |
|  |  | INTM | 0.988 (0.967, 1.01) |
|  | **age≥60** | RERI | -0.023 (-0.055, 0.01) |
|  |  | AP | -0.016 (-0.037, 0.005) |
|  |  | SI | 0.948 (0.89, 1.01) |
|  |  | INTM | 0.972 (0.941, 1.004) |
| **Liver diseases** |  |  |  |
|  | **age<60** | RERI | -0.004 (-0.048, 0.04) |
|  |  | AP | -0.003 (-0.032, 0.026) |
|  |  | SI | 0.992 (0.909, 1.082) |
|  |  | INTM | 0.987 (0.946, 1.03) |
|  | **age≥60** | RERI | 0.04 (-0.021, 0.1) |
|  |  | AP | 0.03 (-0.025, 0.085) |
|  |  | SI | 1.136 (0.745, 1.731) |
|  |  | INTM | 1.033 (0.967, 1.105) |

Adjusted model based on gender, age, LDL-C, HbA1c, TyG index, marry status, educational level, residence, smoking status, drinking status, physical activity, BMI categorization, kidney diseases, dyslipidemia, social activity, live alone. RERI, relative excess risk due to interaction; AP, proportion attributable to interaction; SI, synergy index; INTM, interaction term.

Table S7. Interactive effects of frailty and depression on incident disease stratified in no imputed dataset.

| **Group** | **Term** | **Interactive effect (95% CI)** |
| --- | --- | --- |
| **Digestive diseases** |  |  |
|  | RERI | -0.026 (-0.06, 0.007) |
|  | AP | -0.016 (-0.035, 0.003) |
|  | SI | 0.962 (0.919, 1.007) |
|  | INTM | **0.96 (0.929, 0.992)** |
| **Gastrointestinal diseases** |  |  |
|  | RERI | -0.032 (-0.07, 0.005) |
|  | AP | -0.021 (-0.042, 0.001) |
|  | SI | 0.946 (0.893, 1.003) |
|  | INTM | **0.96 (0.926, 0.995)** |
| **Liver diseases** |  |  |
|  | RERI | -0.013 (-0.081, 0.055) |
|  | AP | -0.007 (-0.045, 0.031) |
|  | SI | 0.983 (0.902, 1.071) |
|  | INTM | 0.972 (0.909, 1.038) |

Adjusted model based on gender, age, LDL-C, HbA1c, TyG index, marry status, educational level, residence, smoking status, drinking status, physical activity, BMI categorization, kidney diseases, dyslipidemia, social activity, live alone. RERI, relative excess risk due to interaction; AP, proportion attributable to interaction; SI, synergy index; INTM, interaction term.

**Table S8.** Decomposition of the total association between depression and the risk of disease into direct and indirect associations mediated by frail, stratified by age.

| Subgroups | Association | | Proportion mediated by frail (%) | Association | | Proportion mediated by frail (%) |
| --- | --- | --- | --- | --- | --- | --- |
|  | Unadjusted indirect HR (95% CI) | Unadjusted direct HR (95% CI) |  | Adjusted indirect HR (95% CI) | Adjusted direct HR (95% CI) |  |
| Digestive diseases |  |  |  |  |  |  |
| Age < 60 years |  |  |  |  |  |  |
| No depression | 1 [Reference] |  |  |  |  |  |
| Depression | 1.115 (1.079-1.153) | 1.481 (1.315-1.668) | 21.7 | 1.095 (1.061-1.13) | 1.434 (1.27-1.619) | 20.1 |
| Age ≥ 60 years |  |  |  |  |  |  |
| No depression | 1 [Reference] |  |  |  |  |  |
| Depression | 1.076 (1.04-1.114) | 1.435 (1.241-1.658) | 16.9 | 1.067 (1.032-1.103) | 1.398 (1.204-1.624) | 16.2 |
| Gastrointestinal diseases |  |  |  |  |  |  |
| Age < 60 years |  |  |  |  |  |  |
| No depression | 1 [Reference] |  |  |  |  |  |
| Depression | 1.117 (1.078-1.158) | 1.522 (1.34-1.729) | 20.9 | 1.095 (1.059-1.132) | 1.441 (1.265-1.641) | 19.9 |
| Age ≥ 60 years |  |  |  |  |  |  |
| No depression | 1 [Reference] |  |  |  |  |  |
| Depression | 1.074 (1.035-1.115) | 1.507 (1.286-1.766) | 14.9 | 1.068 (1.03-1.108) | 1.451 (1.232-1.71) | 15 |
| Liver diseases |  |  |  |  |  |  |
| Age < 60 years |  |  |  |  |  |  |
| No depression | 1 [Reference] |  |  |  |  |  |
| Depression | 1.131 (1.063-1.204) | 1.217 (0.954-1.551) | 38.7 | 1.111 (1.047-1.178) | 1.287 (1.004-1.65) | 29.4 |
| Age ≥ 60 years |  |  |  |  |  |  |
| No depression | 1 [Reference] |  |  |  |  |  |
| Depression | 1.121 (1.061-1.185) | 1.077 (0.818-1.418) | 60.6 | 1.097 (1.038-1.16) | 1.108 (0.835-1.472) | 47.5 |

Adjusted model based on gender, age, LDL-C, HbA1c, TyG index, marry status, educational level, residence, smoking status, drinking status, physical activity, BMI categorization, kidney diseases, dyslipidemia, social activity, live alone.

**Table S9.** Decomposition of the total association between depression and the risk of disease into direct and indirect associations mediated by frail, stratified by sex.

| Subgroups | Association | | Proportion mediated by frail (%) | Association | | Proportion mediated by frail (%) |
| --- | --- | --- | --- | --- | --- | --- |
|  | Unadjusted indirect HR (95% CI) | Unadjusted direct HR (95% CI) |  | Adjusted indirect HR (95% CI) | Adjusted direct HR (95% CI) |  |
| Digestive diseases |  |  |  |  |  |  |
| Male |  |  |  |  |  |  |
| No depression | 1 [Reference] |  |  |  |  |  |
| Depression | 1.117 (1.077-1.159) | 1.308 (1.135-1.507) | 29.2 | 1.11 (1.071-1.151) | 1.255 (1.084-1.453) | 31.5 |
| Female |  |  |  |  |  |  |
| No depression | 1 [Reference] |  |  |  |  |  |
| Depression | 1.073 (1.041-1.107) | 1.556 (1.377-1.759) | 13.8 | 1.066 (1.035-1.098) | 1.538 (1.358-1.743) | 12.9 |
| Gastrointestinal diseases |  |  |  |  |  |  |
| Male |  |  |  |  |  |  |
| No depression | 1 [Reference] |  |  |  |  |  |
| Depression | 1.117 (1.073-1.163) | 1.422 (1.217-1.661) | 24 | 1.115 (1.071-1.161) | 1.351 (1.151-1.586) | 26.6 |
| Female |  |  |  |  |  |  |
| No depression | 1 [Reference] |  |  |  |  |  |
| Depression | 1.068 (1.035-1.103) | 1.522 (1.337-1.733) | 13.6 | 1.065 (1.032-1.099) | 1.498 (1.311-1.711) | 13.5 |
| Liver diseases |  |  |  |  |  |  |
| Male |  |  |  |  |  |  |
| No depression | 1 [Reference] |  |  |  |  |  |
| Depression | 1.13 (1.063-1.202) | 1.015 (0.775-1.328) | 89.2 | 1.106 (1.041-1.176) | 1.011 (0.762-1.342) | 90.2 |
| Female |  |  |  |  |  |  |
| No depression | 1 [Reference] |  |  |  |  |  |
| Depression | 1.143 (1.079-1.21) | 1.393 (1.078-1.801) | 28.7 | 1.104 (1.044-1.168) | 1.456 (1.125-1.884) | 20.9 |

Adjusted model based on gender, age, LDL-C, HbA1c, TyG index, marry status, educational level, residence, smoking status, drinking status, physical activity, BMI categorization, kidney diseases, dyslipidemia, social activity, live alone.

**Table S10.** Decomposition of the total association between frailty and the risk of disease into direct and indirect associations mediated by depression, stratified by age.

| Subgroups | Association | | Proportion mediated by depression (%) | Association | | Proportion mediated by depression (%) |
| --- | --- | --- | --- | --- | --- | --- |
|  | Unadjusted indirect HR (95% CI) | Unadjusted direct HR (95% CI) |  | Adjusted indirect HR (95% CI) | Adjusted direct HR (95% CI) |  |
| Digestive diseases |  |  |  |  |  |  |
| Age < 60 years |  |  |  |  |  |  |
| Robust | 1 [Reference] |  |  |  |  |  |
| Frail | 1.1 (1.065-1.136) | 1.483 (1.319-1.669) | 19.5 | 1.083 (1.051-1.116) | 1.438 (1.272-1.625) | 18 |
| Age ≥ 60 years |  |  |  |  |  |  |
| Robust | 1 [Reference] |  |  |  |  |  |
| Frail | 1.082 (1.044-1.122) | 1.428 (1.213-1.682) | 18.1 | 1.072 (1.035-1.111) | 1.387 (1.174-1.639) | 17.6 |
| Gastrointestinal diseases |  |  |  |  |  |  |
| Age < 60 years |  |  |  |  |  |  |
| Robust | 1 [Reference] |  |  |  |  |  |
| Frail | 1.108 (1.07-1.148) | 1.495 (1.316-1.699) | 20.3 | 1.084 (1.049-1.12) | 1.436 (1.258-1.64) | 18.2 |
| Age ≥ 60 years |  |  |  |  |  |  |
| Robust | 1 [Reference] |  |  |  |  |  |
| Frail | 1.096 (1.052-1.141) | 1.42 (1.184-1.702) | 20.7 | 1.082 (1.04-1.125) | 1.407 (1.17-1.693) | 18.8 |
| Liver diseases |  |  |  |  |  |  |
| Age < 60 years |  |  |  |  |  |  |
| Robust | 1 [Reference] |  |  |  |  |  |
| Frail | 1.047 (0.985-1.113) | 1.583 (1.244-2.014) | 9.1 | 1.06 (1.001-1.123) | 1.535 (1.2-1.964) | 12 |
| Age ≥ 60 years |  |  |  |  |  |  |
| Robust | 1 [Reference] |  |  |  |  |  |
| Frail | 1.013 (0.954-1.077) | 1.8 (1.306-2.479) | 2.2 | 1.022 (0.963-1.085) | 1.613 (1.159-2.246) | 4.4 |

Adjusted model based on gender, age, LDL-C, HbA1c, TyG index, marry status, educational level, residence, smoking status, drinking status, physical activity, BMI categorization, kidney diseases, dyslipidemia, social activity, live alone.

**Table S11.** Decomposition of the total association between frailty and the risk of disease into direct and indirect associations mediated by depression, stratified by sex.

| Subgroups | Association | | Proportion mediated by depression (%) | Association | | Proportion mediated by depression (%) |
| --- | --- | --- | --- | --- | --- | --- |
|  | Unadjusted indirect HR (95% CI) | Unadjusted direct HR (95% CI) |  | Adjusted indirect HR (95% CI) | Adjusted direct HR (95% CI) |  |
| Digestive diseases |  |  |  |  |  |  |
| Male |  |  |  |  |  |  |
| Robust | 1 [Reference] |  |  |  |  |  |
| Frail | 1.056 (1.022-1.091) | 1.511 (1.321-1.728) | 11.6 | 1.045 (1.012-1.08) | 1.508 (1.311-1.736) | 9.7 |
| Female |  |  |  |  |  |  |
| Robust | 1 [Reference] |  |  |  |  |  |
| Frail | 1.111 (1.075-1.148) | 1.384 (1.211-1.581) | 24.4 | 1.103 (1.068-1.14) | 1.373 (1.195-1.576) | 23.6 |
| Gastrointestinal diseases |  |  |  |  |  |  |
| Male |  |  |  |  |  |  |
| Robust | 1 [Reference] |  |  |  |  |  |
| Frail | 1.074 (1.034-1.115) | 1.511 (1.298-1.758) | 14.8 | 1.061 (1.022-1.101) | 1.54 (1.314-1.804) | 12 |
| Female |  |  |  |  |  |  |
| Robust | 1 [Reference] |  |  |  |  |  |
| Frail | 1.105 (1.068-1.144) | 1.355 (1.177-1.561) | 24.8 | 1.096 (1.059-1.134) | 1.366 (1.18-1.581) | 22.7 |
| Liver diseases |  |  |  |  |  |  |
| Male |  |  |  |  |  |  |
| Robust | 1 [Reference] |  |  |  |  |  |
| Frail | 1.005 (0.949-1.064) | 1.604 (1.255-2.05) | 1.1 | 1.006 (0.949-1.065) | 1.49 (1.151-1.928) | 1.4 |
| Female |  |  |  |  |  |  |
| Robust | 1 [Reference] |  |  |  |  |  |
| Frail | 1.079 (1.012-1.151) | 1.897 (1.396-2.578) | 10.6 | 1.092 (1.026-1.163) | 1.682 (1.223-2.314) | 14.5 |

Adjusted model based on gender, age, LDL-C, HbA1c, TyG index, marry status, educational level, residence, smoking status, drinking status, physical activity, BMI categorization, kidney diseases, dyslipidemia, social activity, live alone.

**Table S12.** Decomposition of the total association between depression and the risk of disease into direct and indirect associations mediated by frailty in no imputed dataset.

| Subgroups | Association | | Proportion mediated by frail (%) |
| --- | --- | --- | --- |
|  | Adjusted indirect HR (95% CI) | Adjusted direct HR (95% CI) |  |
| Digestive diseases |  |  |  |
| No depression | 1 [Reference] |  |  |
| Depression | 1.098 (1.072-1.125) | 1.464 (1.335-1.605) | 19.8 |
| Gastrointestinal diseases |  |  |  |
| No depression | 1 [Reference] |  |  |
| Depression | 1.097 (1.069-1.126) | 1.518 (1.374-1.676) | 18.2 |
| Liver diseases |  |  |  |
| No depression | 1 [Reference] |  |  |
| Depression | 1.133 (1.085-1.183) | 1.155 (0.962-1.386) | 46.5 |

Adjusted model based on gender, age, LDL-C, HbA1c, TyG index, marry status, educational level, residence, smoking status, drinking status, physical activity, BMI categorization, kidney diseases, dyslipidemia, social activity, live alone.

**Table S13.** Decomposition of the total association between frailty and the risk of disease into direct and indirect associations mediated by depression in no imputed dataset.

| Subgroups | Association | | Proportion mediated by depression (%) |
| --- | --- | --- | --- |
|  | Adjusted indirect HR (95% CI) | Adjusted direct HR (95% CI) |  |
| Digestive diseases |  |  |  |
| Robust | 1 [Reference] |  |  |
| Frail | 1.093 (1.067-1.119) | 1.452 (1.321-1.596) | 19.2 |
| Gastrointestinal diseases |  |  |  |
| Robust | 1 [Reference] |  |  |
| Frail | 1.103 (1.074-1.133) | 1.446 (1.304-1.604) | 21 |
| Liver diseases |  |  |  |
| Robust | 1 [Reference] |  |  |
| Frail | 1.032 (0.988-1.078) | 1.678 (1.387-2.03) | 5.7 |
| Adjusted model based on gender, age, LDL-C, HbA1c, TyG index, marry status, educational level, residence, smoking status, drinking status, physical activity, BMI categorization, kidney diseases, dyslipidemia, social activity, live alone. | | | |

**Figure S13.** Incremental predictive value (ROC curve) of depression and frailty index beyond other factors.

A. Digestive diseases; B. Gastrointestinal diseases; C. Liver diseases.

**Table S14.** Incremental predictive value (IDI and NRI) of depression and frailty index beyond other factors.

| Models | IDI (95% CI) | *P*_NRI_ | NRI (95% CI) | *P*_IDI_ |
| --- | --- | --- | --- | --- |
| **Digestive diseases** |  |  |  |  |
| Covariate^a^ | Reference |  | Reference |  |
| Covariate + Depression + Frailty | 0.02(0.003, 0.025) | <0.001 | 0.141(0.025, 0.167) | <0.001 |
| **Gastrointestinal diseases** |  |  |  |  |
| Covariate | Reference |  | Reference |  |
| Covariate + Depression + Frailty | 0.021(0.004, 0.021) | <0.001 | 0.14(0.031, 0.148) | <0.001 |
| **Liver diseases** |  |  |  |  |
| Covariate | Reference |  | Reference |  |
| Covariate + Depression + Frailty | 0.004(0, 0.005) | <0.001 | 0.136(0.009, 0.168) | <0.001 |

Adjusted model based on gender, age, LDL-C, HbA1c, TyG index, marry status, educational level, residence, smoking status, drinking status, physical activity, BMI categorization, kidney diseases, dyslipidemia, social activity, live alone. NRI, net reclassification improvement; IDI, integrated discrimination improvement; LR, likelihood ratio test.

**Table S15.** E-values of triglyceride-glucose index and body mass index with digestive diseases.

| **Subgroups** | **E-value (95% CI)** | | |
| --- | --- | --- | --- |
|  | **Digestive diseases** | **Gastrointestinal diseases** | **Liver diseases** |
| Frailty |  |  |  |
| Robust | Reference | Reference | Reference |
| Pre-Frail | 2.66 (2.01, 3.39) | 2.60 (1.92, 3.41) | 2.77 (1.54, 4.46) |
| Frail | 3.78 (2.52, 5.47) | 4.27 (2.83, 6.28) | 3.62 (1.54, 7.24) |
| Depression |  |  |  |
| No | Reference | Reference | Reference |
| Yes | 2.71 (2.10, 3.43) | 2.62 (1.99, 3.39) | 2.43 (1.31, 3.82) |

Adjusted model based on gender, age, LDL-C, HbA1c, TyG index, marry status, educational level, residence, smoking status, drinking status, physical activity, BMI categorization, kidney diseases, dyslipidemia, social activity, live alone.
